# Evidence that neutrophils do not promote *Echis carinatus* venom-induced tissue destruction

**DOI:** 10.1038/s41467-018-04688-6

**Published:** 2018-06-13

**Authors:** Julien Stackowicz, Bianca Balbino, Biliana Todorova, Ophélie Godon, Bruno Iannascoli, Friederike Jönsson, Pierre Bruhns, Laurent L. Reber

**Affiliations:** 1 Unit of Antibodies in Therapy and Pathology, Department of Immunology, Institut Pasteur, Paris, 75015 France; 2 INSERM U1222, Paris, France; 3 Université Pierre et Marie Curie, Paris, 75252 France; 4 Department of Biology, École Normale Supérieure Paris-Saclay, 94230 Cachan, France

## Introduction

Katkar et al.^1^ propose that neutrophils can mediate tissue damage induced by venom of the saw-scaled viper (*Echis carinatus*) by generating neutrophil extracellular traps (NETs), which can entrap venom toxins at the injection site, thereby accelerating local tissue destruction but protecting from systemic damage. Each year, snakebites are responsible for more than 400,000 amputations and 125,000 deaths worldwide^2^. Together, the Indian cobra (*Naja naja*), the krait (*Bungarus caerulaeus*), the Russell’s viper (*Daboia russelii*), and *E. carinatus* compose the group of snakes referred to as the “Big Four”, which account for the majority of snakebite-related deaths in India^3^. *E*. *carinatus* venom (ECV) induces particularly strong tissue destruction at the bite site, but the cellular and molecular mechanisms regulating this tissue damage are unclear.

**Fig. 1:**
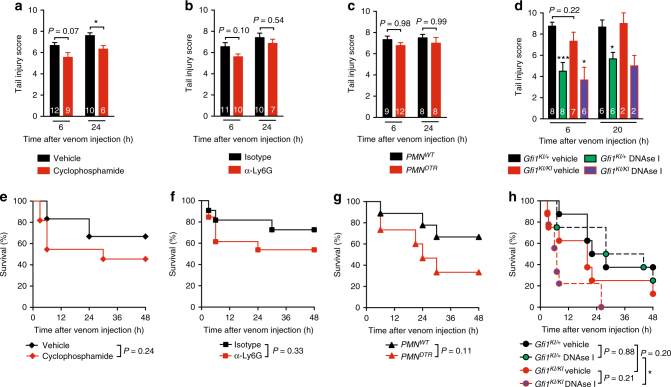
ECV-induced tail injury and mortality in neutrophil-deficient mice and mice treated with cyclophosphamide or DNAse I. **a**–**h** Tail injury scores (**a**–**d**) and survival (percentage of live animals) after injection of *E*. *carinatus sochukeri* venom (3 mg/kg) (**e**–**h**) in mice treated with cyclophosphamide or vehicle (PBS) (*n* = 12/group) (**a**, **e**), neutrophil-depleting anti-Ly6G antibodies or isotype control antibodies (*n* = 12/group) (**b**, **f**), and in neutrophil-depleted diphtheria toxin (DT)-treated *PMN*^*DTR*^ mice (*n* = 15) or DT-treated *PMN*^*WT*^ littermate controls (*n* = 9) (**c**, **g**), and in neutrophil-deficient *Gfi1*^*KI/KI*^ mice or neutrophil-sufficient *Gfi1*^*KI*/+^ littermates injected with *E*. *carinatus sochukeri* venom (3 mg/kg) alone or together with 500 U DNAse I (*n* = 8–9/group) (**d**, **h**). Data are pooled from two or three independent experiments. White numbers in **a**–**d** indicate number of live animals per group at each time point. *P < 0.05 and ***P < 0.01 by two-tailed Mann–Whitney *U* test (**a**, **d**) or Mantel–Cox log-rank test (**e**–**h**)

As part of this study, Katkar et al. report that treatment of mice with cyclophosphamide reduces tissue damage induced by *E*. *carinatus carinatus* (South Indian saw-scaled viper) venom, an effect they attribute to deficiency of neutrophils in cyclophosphamide-treated mice^1^. However, when we treat mice with cyclophosphamide before injecting venom from *E*. *carinatus sochureki* in the tail, we find that cyclophosphamide has very little effect on mortality and venom-induced tissue destruction, as assessed by the same tail injury scoring method used by Katkar et al.^1^ (Fig. 1a, e). This distinction might reflect differences in the types and amounts of venoms used, so we confirm our results using venom from two other *E*. *carinatus* subspecies: *E*. *carinatus multisquamatus* and *E*. *carinatus pyramidum*. Again, treatment with cyclophosphamide does not reduce tail injury and has no effect on mortality after injection of venoms from these two subspecies of *E*. *carinatus* (Supplementary Fig. 1). As expected, treatment with cyclophosphamide induces strong neutropenia (Supplementary Fig. 2a), but this broad immunosuppressive drug also reduces the number of circulating monocytes, T cells, and B cells (Supplementary Fig. 2b–d), and the spleen weight by 40% (Supplementary Fig. 2e, f). Therefore, we do not think the effect of this pleiotropic drug in the article by Katkar et al.^1^ is attributable to neutrophils.

Because cyclophosphamide suppresses many cell populations, and different batches of the drug could differentially affect degree of neutropenia (which might also contribute to the differences between our results and those of Katkar et al.^1^), we reevaluate the role of neutrophils in response to ECV using antibody or genetic approaches to deplete neutrophils in mice. Similar to our results with cyclophosphamide, treatment of mice with neutrophil-depleting anti-Ly6G antibodies has no effect on *E*. *carinatus sochureki* venom-induced tail injury (Fig. 1b) or mortality (Fig. 1f). We also use *PMN*^*DTR*^ mice, which express the diphtheria toxin (DT) receptor specifically on neutrophils, and in which >95% of circulating and tissue neutrophils can be depleted upon injection of DT^4^. ECV-induced tail injury and mortality in DT-treated neutrophil-deficient *PMN*^*DTR*^ mice compared to DT-treated neutrophil-sufficient *PMN*^*WT*^ littermates is not affected (Fig. 1c, g). The lack of effect after antibody-mediated or DT-mediated neutropenia induction is not due to persistence of circulating neutrophils in blood (Supplementary Figs. 3a, b and 4a, b) or at the venom injection site (Supplementary Figs. 3c and 4c) 5 h after venom injection, a time point at which important tail injury already occurs. We further confirm our results using mice deficient for growth factor independence-1 (Gfi1), a transcription repressor that enables neutrophil differentiation^5^. Indeed, similar tail injury and mortality occurred after ECV injection in neutrophil-deficient *Gfi1*^*KI/KI*^ mice^6^ compared to neutrophil-sufficient *Gfi1*^*KI/+*^ littermates (Fig. 1d, h). At a lower dose of venom, both tail injury 8 h after venom injection and mortality are increased in neutrophil-deficient *Gfi1*^*KI/KI*^ mice as compared to *Gfi1*^*KI/+*^ mice (Supplementary Fig. 5a, b). These responses might be a consequence of neutropenia in *Gfi1*^*KI/KI*^ mice (Supplementary Fig. 5c) or of other phenotypic abnormalities associated with Gfi1 deficiency^5^.

Interestingly, Katkar et al.^1^ report that treatment of mice with DNAse I, which can degrade extracellular DNA, reduces tissue damage at the site of venom injection^1^. The authors suggest that these effects are due to the clearance of NETs by DNAse I. We confirm that treatment with DNAse I can reduce ECV-induced tail injury (Fig. 1d). However, DNAse I has similar effects in both neutrophil-sufficient *Gfi1*^*KI/+*^ and neutrophil-deficient *Gfi1*^*KI/KI*^ mice (Fig. 1d). Katkar et al.^1^ also report increased ECV-induced mortality of mice treated with DNAse I^1^. We too observe an increase in mortality after ECV injection of DNAse I-treated mice, but only with neutropenic *Gfi1*^*KI/KI*^ mice and not neutrophil-sufficient *Gfi1*^*KI/+*^ littermates (Fig. 1h).

Altogether, our data indicate that neutrophils do not promote ECV-induced tissue damage at the site of venom injection. Our data using a low dose of venom in *Gfi*^*KI/KI*^ mice even suggest that neutrophils might help to reduce tail injury. However, using different approaches, we confirm the finding by Katkar et al.^1^ that neutropenic mice are more susceptible to mortality after ECV injection. While treatment with DNAse I might be of great interest to reduce local tissue damage induced by the venom, the effects of the drug in our study are probably not a result of the degradation of NETs. Some toxins in ECV have strong cytotoxic activities that cause broad cell destruction and tissue necrosis^7^, and favor DNA release at the injection site. This DNA may trap venom toxins, a mechanism proposed by Katkar et al.^1^, but we propose that neutrophil-derived DNA might not be essential for this protective mechanism, and that protection could be conferred by DNA released by a broad range of necrotic cells.

It is important to note that several factors might contribute to the difference in results between our study and that of Katkar et al.^1^. Among those differences, our studies use venoms from different subspecies of *E*. *carinatus* (Fig. 1 and Supplementary Fig. 1). Neutrophils and NETs might contribute differently to protection against subspecies venoms. In addition, differences in the microbiological environment between mice in the two animal facilities might contribute; commensal bacteria have been shown to regulate neutrophil aging, and aged neutrophils are more prone to make NETs^8^.

In conclusion, our study confirms that treatment with DNAse I can reduce tissue damage induced by ECV, which may have important clinical implications, but we highlight the fact that more research is needed before definitive conclusions can be made on the role of neutrophils and NETs in this process.

## Methods

### Mice

RjOrl:SWISS mice were purchased from Janvier Labs. *PMN*^*DTR*^ mice (*MRP8-Cre; iDTR*^*fl*/+^) on a C57BL/6 background were described previously^4^. Neutrophil-deficient *Gfi1*^*KI/KI*^ mice on a C57BL/6 background were provided by T. Moroy (Montreal University, Montreal, QC, Canada)^6^, and we used neutrophil-sufficient *Gfi1*^*KI*/+^ as littermate controls. Mice were bred and maintained at the Institut Pasteur animal facility, or housed in our animal facility for at least 7 days before starting experiments. We used age- and sex-matched mice for all experiments. All animal experimentations were conducted with the specific approval of the CETEA ethics committee number 89 (Institut Pasteur, Paris, France) under #dap160072.

### Venom-induced mouse tail tissue destruction and lethality

Lyophilized *E. carinatus sochureki* venom was purchased from Kentucky Reptile Zoo (USA), and used for most experiments (Fig. 1 and Supplementary Figs. 2–5). In Supplementary Fig. 1, we used lyophilized *E. carinatus multisquamatus* and *E. carinatus pyramidum* venoms purchased from Latoxan (France). All venoms were resuspended in sterile phosphate-buffered saline (PBS) at a concentration of 100 mg/mL and stored at −20 °C. *E. carinatus* venom was administered at the indicated dose subcutaneously 3 cm distal to the base of the tail in a volume of 25 μL. Local damages were assessed at different time points according to a 10-point scale described by Katkar et al.^1^ 0: no visible injury; 1: edema; 2: edema with minor hemorrhage; 4: edema with hemorrhage causing <25% tail discoloration; 6: edema and major hemorrhage or wound causing 25–50% tail discoloration; 8: edema and major hemorrhage or wound causing 50–75% tail discoloration; and 10: edema and major hemorrhage or wound causing more than 75% tail discoloration. Mice were observed for mortality at least four times daily. Mice that were clearly moribund were euthanized by CO_2_ inhalation.

### Treatment with cyclophosphamide

RjOrl:SWISS mice were treated with cyclophosphamide as described by Katkar et al.^1^. Mice were injected intraperitoneally (i.p.) with cyclophosphamide (Sigma-Aldrich) twice (150 mg/kg on day one and 100 mg/kg on day four, in 500 μL PBS) and *E. carinatus* venom was injected 24 h after the last injection of cyclophosphamide. In the experiments presented in Supplementary Fig. 2, groups of mice were sacrificed 24 h after the last injection of cyclophosphamide to assess effects of the drug on neutrophils and other cell populations in the blood by flow cytometry.

### Treatment with DNAse I

Recombinant DNAse I was purchased from Roche Diagnostics. DNAse I was co-incubated with *E. carinatus* venom for 30 min and co-injected in mice at a final dose of 500 U in 25 μL PBS.

### Antibody-mediated neutrophil depletion

Neutrophil-depleting anti-Ly6G antibodies were purified in our laboratory (using protein G columns) from culture supernatants of the NIMP-R14 hybridoma provided by Dr. Claude Leclerc (Institut Pasteur, France). Mice were injected i.p. with anti-Ly6G antibodies (NIMP-R14), or rat IgG2b isotype control antibodies (clone LTF2; BioXcell; each at 300 μg in 200 μL PBS) 24 h before, 2 h before, and 24 h after injection of venom. The efficiency of neutrophil depletion was verified by immunochemistry at the site of venom injection and by flow cytometry in the blood and spleen 5 h post injection. These data are presented in a Supplementary Fig. 3.

### DT-mediated neutrophil depletion

*PMN*^*DTR*^ mice were injected i.p. with DT (500 ng in 200 μL PBS; Sigma-Aldrich) to selectively deplete neutrophils^4^. DT injections were performed 24 h before, 2 h before, and 24 h after injection of venom. We used DT-treated *PMN*^*WT*^ littermates as neutrophil-sufficient control mice. The efficiency of neutrophil depletion was verified by immunochemistry at the site of venom injection and by flow cytometry in the blood and spleen 5 h post injection. These data are presented in a Supplementary Fig. 4.

### Flow cytometry

We used flow cytometry to identify and enumerate immune cell populations in peripheral blood and spleen. Briefly, red blood cells were lysed by treatment with red blood cell lysis buffer (BD Biosciences). Cells were stained with a combination of antibodies on ice for 30 min. Immune cell populations were identified as follows: neutrophils (CD11b^+^; Ly6G^+^); monocytes (CD11b^+^; Ly6G^−^); T cells (CD3ε^+^; B220^−^); and B cells (CD3ε^-^; B220^+^). Gating strategies are detailed in Supplementary Fig. 6. Antibodies used were as follows: Ly6G-BV421 (1A8); CD11b-FITC (M1/70); CD3ε-PE (145-2C11); or B220-APCCy7 (RA3-6B2). All antibodies were purchased from BD Biosciences. Data were acquired using a MACSQuant (Miltenyi) flow cytometer and analyzed with FlowJo software (TreeStar). Dead cells (identified by staining with propidium iodide) were not included in the analysis.

### Immunofluorescence

In experiments presented in Supplementary Figs. 3, 4, mice were euthanized by CO_2_ inhalation 5 h after injection of venom for histological analysis of tail tissues. A skin biopsy was performed at the site of venom injection, and processed for histological analysis. Briefly, skin biopsies were fixed overnight with 1% paraformaldehyde, dehydrated with increasing sucrose concentrations (10, 20, and 30%), and embedded in optimal cutting temperature compound. Eight-micrometer-thick sections were prepared using a cryostat. Slides were incubated 1 h in permeabilization buffer (eBiosciences) containing 0.5% bovine serum albumin. Slides were stained with antibodies directed against myeloperoxidase (MPO; 4 μg/mL, R&D, AF3667 in permeabilization buffer overnight at 4 °C in the dark). For the detection of MPO, slides were washed three times in permeabilization buffer and incubated for 1 h in the dark with Alexa647-coupled donkey anti-goat IgG (10 μg/mL, Molecular Probes). Slides were washed three times and mounted with coverslips using Prolong Gold antifade reagent (Invitrogen), which contains 4′,6-diamidino-2-phenylindole to stain DNA. Samples were imaged by using a confocal microscope Leica SP5, ×40 objective, and analyzed using ImageJ software. Controls were stained with secondary antibodies only, which revealed no unspecific staining (data not shown).

### Statistical analyses

Data are presented as means + SEM. Differences between groups were assessed for statistical significance using Mantel–Cox or unpaired Mann–Whitney *U* tests, where applicable. *P* values < 0.05 are considered statistically significant.

### Data availability

The data that support the findings of this study are available from the corresponding author upon request.

## Electronic supplementary material

Supplementary Information
