# Supplementary material for: Evidence that neutrophils do not promote Echis carinatus venom-induced tissue destruction
Source: Nat Commun. 2018 Jun 13;9:2304. doi: 10.1038/s41467-018-04688-6 (PMC5998045; doi:10.1038/s41467-018-04688-6)
Supplement: Supplementary file 1 — Supplementary Information [file 41467_2018_4688_MOESM1_ESM.pdf]

Supplementary Information

‘Correspondence: Evidence that neutrophils do not promote *Echis carinatus* venom-induced tissue destruction’

Stackowicz et al.

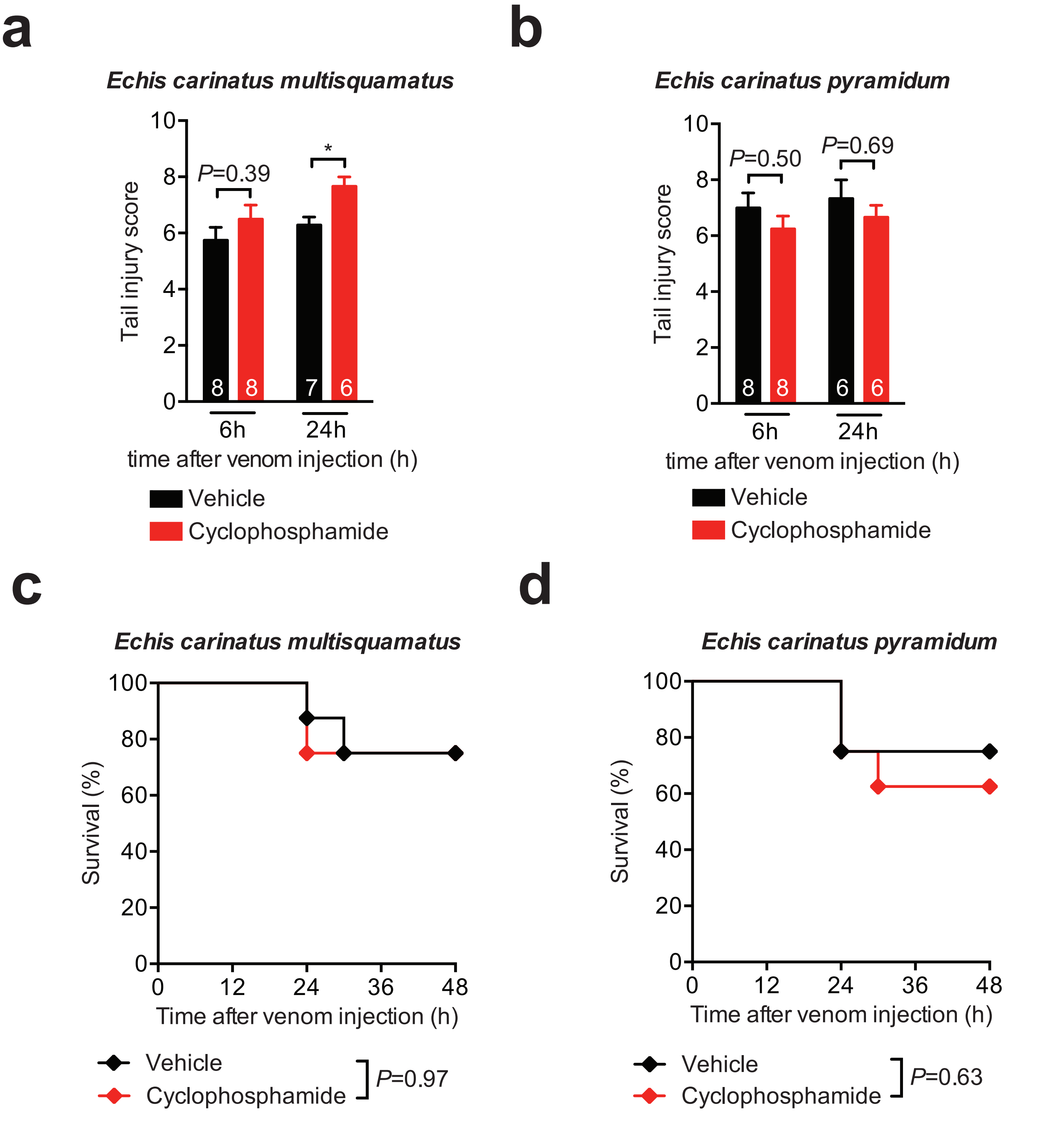

Supplementary Figure 1. Effect of cyclophosphamide in mice injected with *Echis carinatus multisquamatus* venom (ECMV), or *Echis carinatus pyramidum* venom (ECPV). RjOrl:SWISS mice were treated with cyclophosphamide as described by Katkar *et al.*[3]. Mice were injected i.p. with cyclophosphamide twice (150 mg/kg on day one and 100 mg/kg on day four, in 500 μL PBS). Control mice were injected with vehicle only (PBS). On day 5, mice were injected in the tail with 3 mg/kg ECMV or ECPV, as indicated. (**a-d**) Tail injury scores (**a** & **b**) and survival (percentage of live animals) (**c** & **d**) after injection of venom (3 mg/kg) in mice pre-treated with cyclophosphamide or vehicle (PBS) (n=8 per group). Numbers of live animals per group in **a** & **b** are indicated in white. Data are pooled from two independent experiments. *, $P < 0.05$; **, $P < 0.01$; ***, $P < 0.001$ by two-tailed Mann–Whitney U test (**a** & **b**) or Mantel–Cox log-rank test (**c** & **d**).

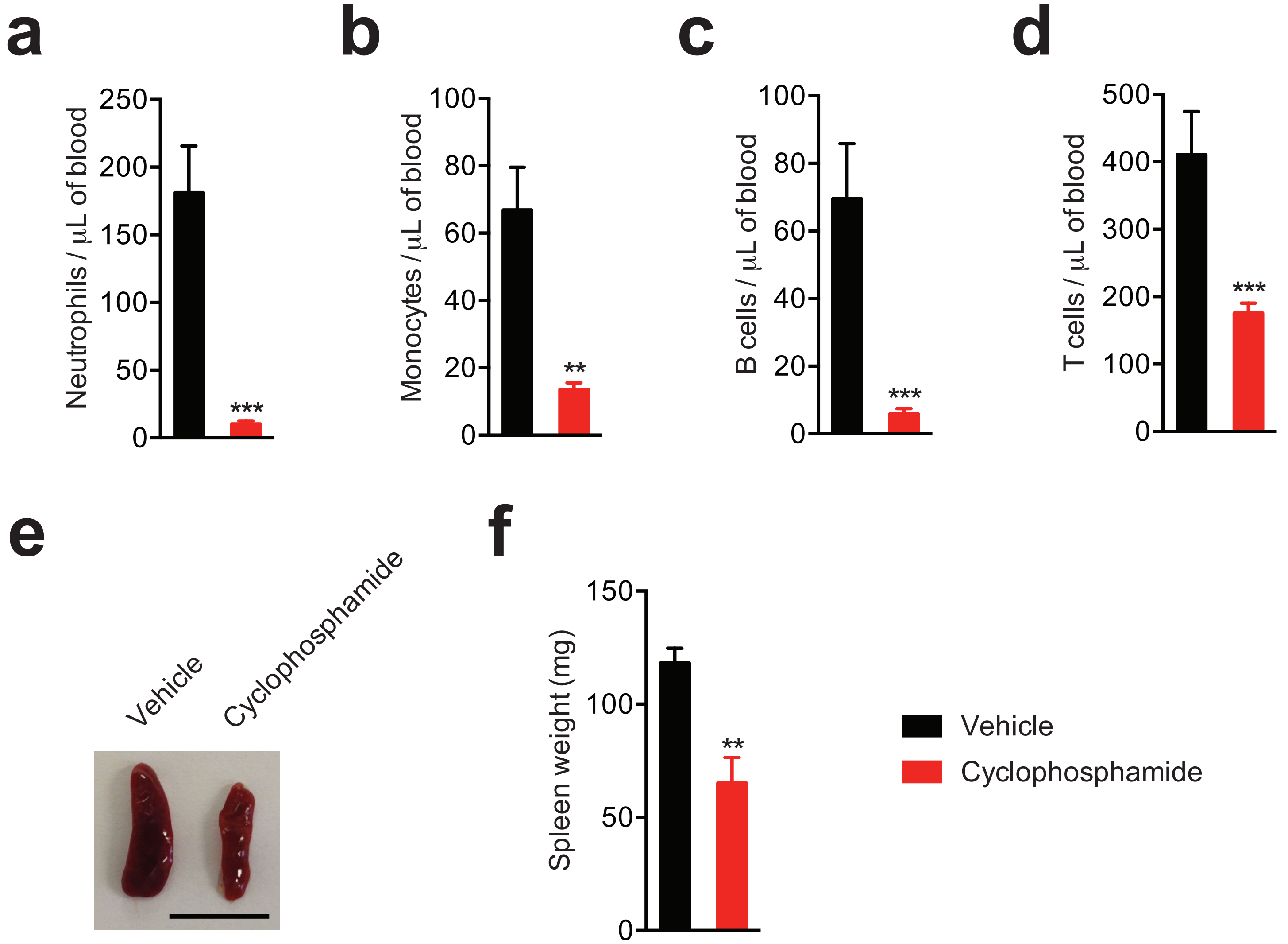

Supplementary Figure 2. Effects of cyclophosphamide on various blood immune cell types and spleen weight. RjOrl:SWISS mice were treated with cyclophosphamide as described by Katkar *et al.*[3]. Mice were injected i.p. with cyclophosphamide twice (150 mg/kg on day one and 100 mg/kg on day four, in 500 µL PBS). Control mice were injected with vehicle only (PBS). Mice were sacrificed on day 5 for analysis of blood immune cell populations and spleen weights. (**a-d**) Numbers of blood $CD11b^+$ $Ly6G^+$ neutrophils (**a**), $CD11b^+$ $Ly6G^-$ monocytes (**b**), $B220^+$ $CD3\varepsilon^-$ B cells (**c**) and $B220^-$ $CD3\varepsilon^+$ T cells (**d**) in mice treated with cyclophosphamide or vehicle (PBS). (**e** & **f**) Representative picture of spleens (**e**) and quantification of spleen weight (**e**) in mice treated with cyclophosphamide or vehicle (PBS). Data are presented as mean + SEM, and pooled from two independent experiments with a total of 6 (**f**) or 8 (**a-d**) mice per group. **, $P < 0.01$; ***, $P < 0.001$ by two-tailed Mann-Whitney *U* test. Scale bar in **e**: 1 cm.

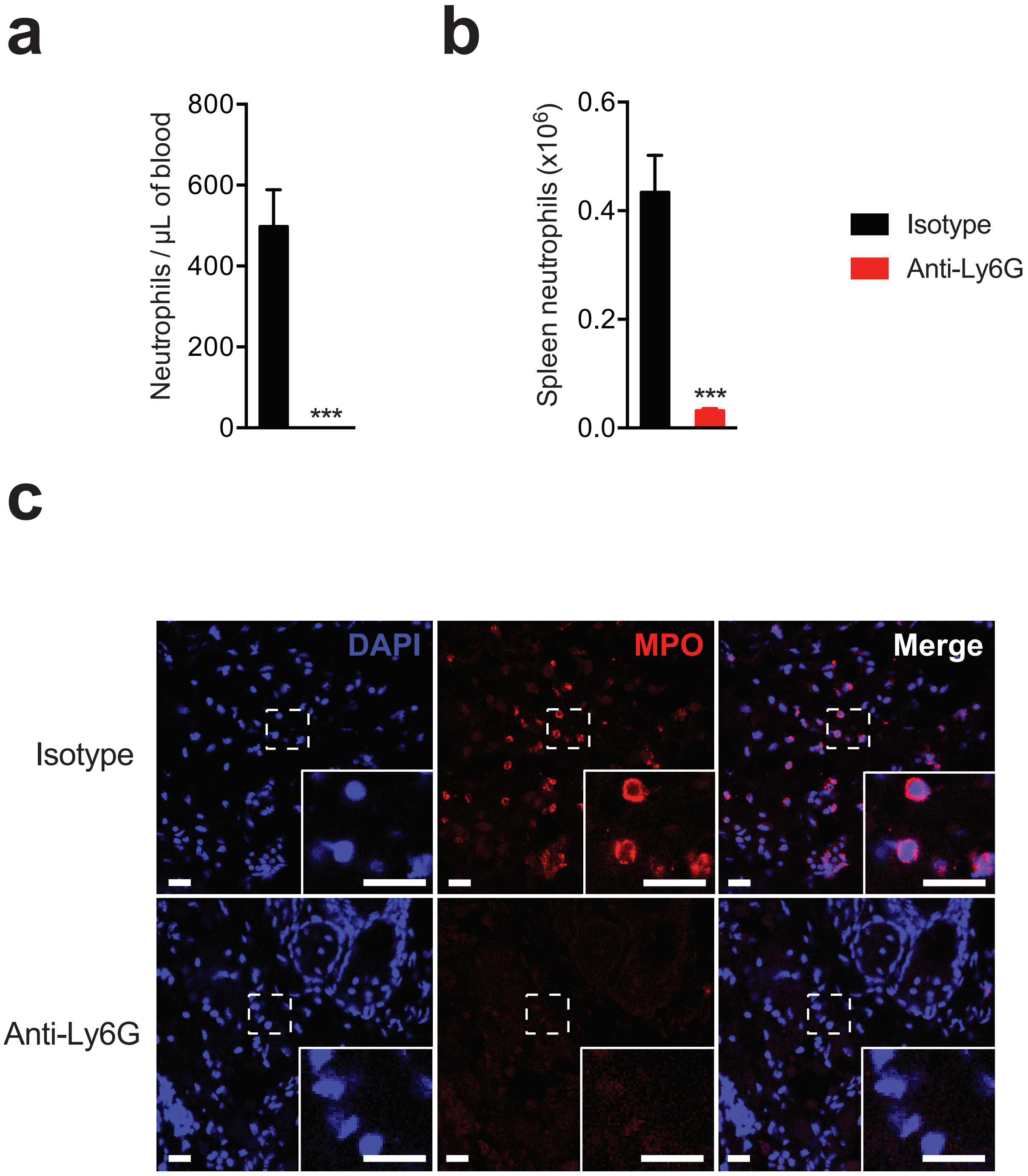

Supplementary Figure 3. Levels of neutrophils 5 hours after injection of *Echis carinatus* venom in mice pre-treated with neutrophil-depleting anti-Ly6G antibodies or isotype control antibodies. (a-c) RjOrl:SWISS mice were treated i.p. with anti-Ly6G antibodies (clone NIMP-R14) or isotype control antibodies (each at 300 µg in 200 µL PBS) 24 h and 2 h before injection of 3 mg/kg *Echis carinatus sochukeri* venom. Mice were sacrificied 5 h after venom injection, and levels of Ly6G$^+$ CD11b$^+$ neutrophils were quantified by flow cytometry in the blood (**a**) and spleen (**b**). Data are presented as mean + SEM, and pooled from two independent experiments with a total of 7 or 8 mice per group. ***, $P < 0.001$ by two-tailed Mann–Whitney U test. (**c**) Confocal microscopy analyses of tail tissues collected after 5 h at the site of venom injection. Representative DNA staining (DAPI), together with myeloperoxidase (MPO) staining. Insets show enlargements of boxed areas. Scale bars, 20 µm.

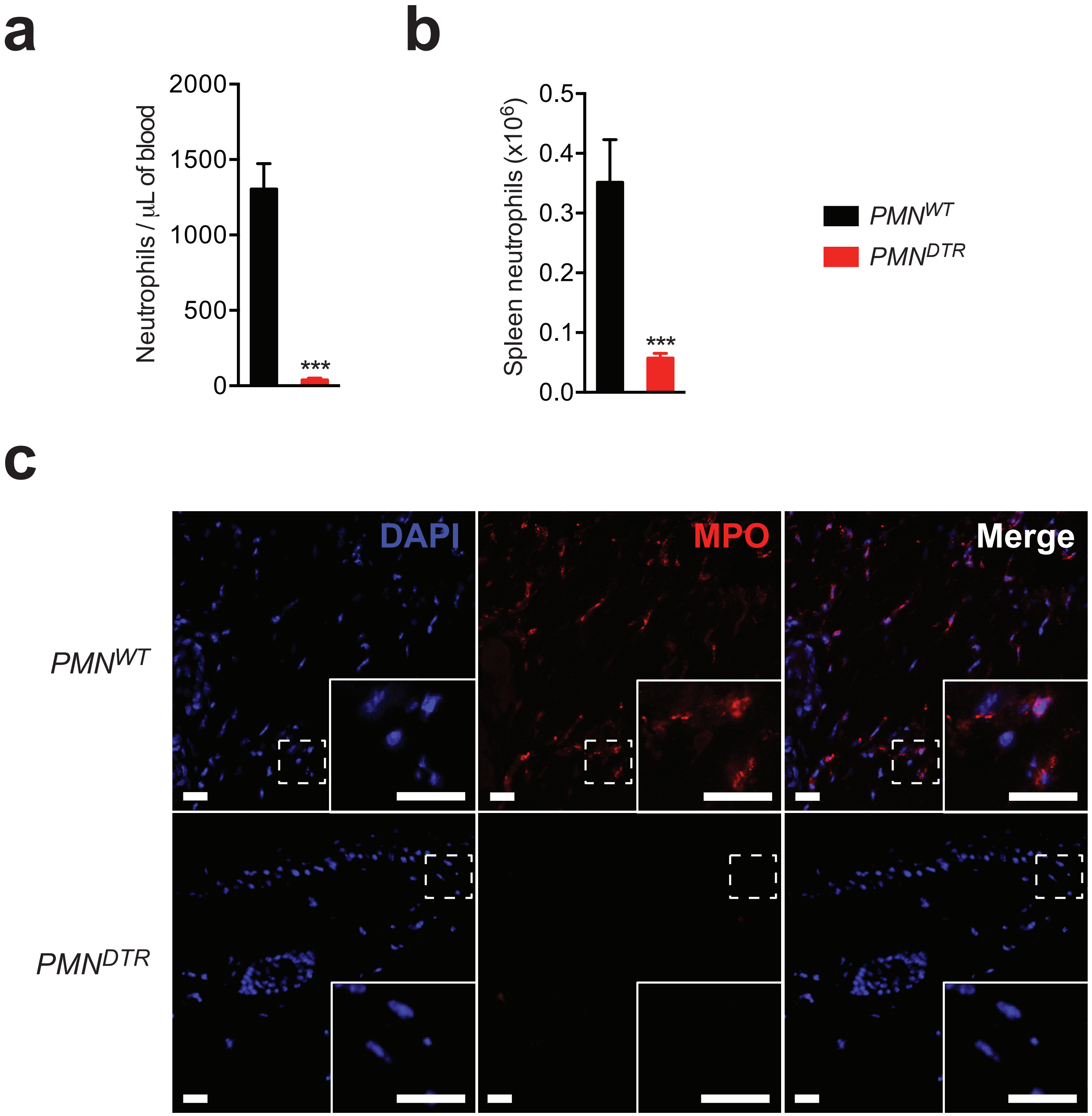

Supplementary Figure 4. Levels of neutrophils 5 hours after injection of *Echis carinatus* venom in DT-treated *PMN*^{DTR} and *PMN*^{WT} mice. (**a-c**) *PMN*^{DTR} and *PMN*^{WT} mice were treated i.p. with 500 ng diphtheria toxin (DT) 24 h and 2 h before injection of 3 mg/kg *Echis carinatus sochukeri* venom. Mice were sacrificied 5 h after venom injection, and levels of Ly6G^{+} CD11b^{+} neutrophils were quantified by flow cytometry in the blood (**a**) and spleen (**b**). Data are presented as mean + SEM, and pooled from two independent experiments with a total of 7 or 8 mice per group. ***, $P < 0.001$ by two-tailed Mann–Whitney U test. (**c**) Confocal microscopy analyses of tail tissues collected after 5 h at the site of venom injection. Representative DNA staining (DAPI), together with myeloperoxidase (MPO) staining. Insets show enlargements of boxed areas. Scale bars, 20 µm.

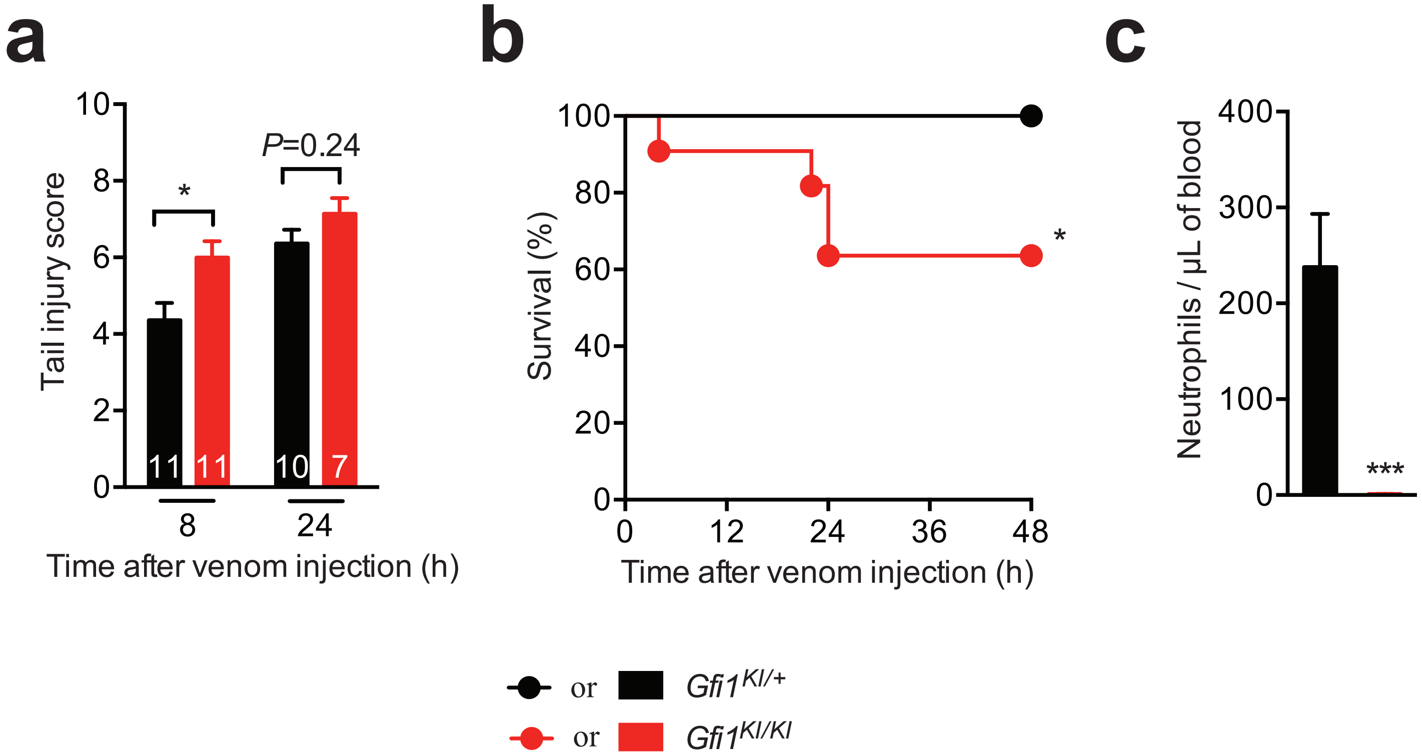

Supplementary Figure 5. Tail injury score, mortality and neutrophil levels in neutrophil-deficient *Gfi1*$^{KI/KI}$ mice and neutrophil-sufficient *Gfi1*$^{KI/+}$ littermates after injection of 1 mg/kg *Echis carinatus* venom. (**a** & **b**) Tail injury scores (**a**) and survival (percentage of live animals) (**b**) after injection of 1 mg/kg *Echis carinatus sochukeri* venom in *Gfi1*$^{KI/KI}$ and *Gfi1*$^{KI/+}$ mice. (**c**) Levels of blood Ly6G$^+$ CD11b$^+$ neutrophils 24 h after injection of venom. Results in **a** & **c** are represented as means + SEM. Data in **a-c** are pooled from two independent experiments with a total of 7 (**c**) or 11 (**a** & **b**) mice per group. White numbers in **a** indicate numbers of live animals per group at each time-point. *, $P < 0.05$; ***, $P < 0.001$ by two-tailed Mann–Whitney *U* test (**a** & **c**) or Mantel-Cox log-rank test (**b**).

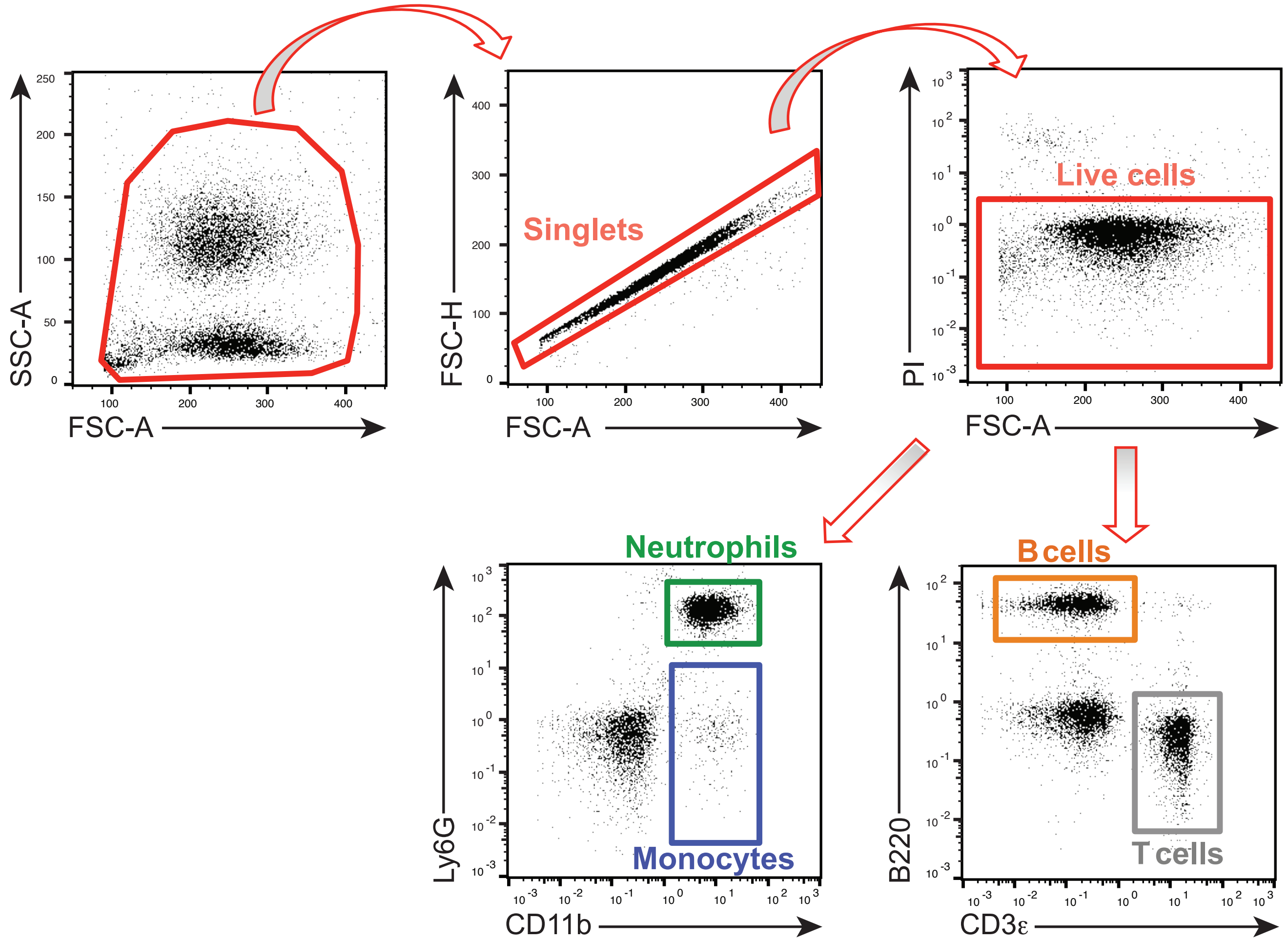

Supplementary Figure 6. Flow cytometry gating strategies. Leukocytes were selected using a side scatter area (SSC-A) *vs*. forward scatter area (FSC-A) density plot. A FSC height (FSC-H) *vs*. FSC-A density plot was used to exclude doublets, and dead cells were excluded based on staining with propidium iodide (PI). Blood immune cell populations were identified among live cells as follows: neutrophils ($CD11b^+$ $Ly6G^+$), monocytes ($CD11b^+$ $Ly6G^-$), B cells ($B220^+$ $CD3\varepsilon^-$) and T cells ($B220^-$ $CD3\varepsilon^+$).
